# Large-Scale Synthesis of Palladium Concave Nanocubes with High-Index Facets for Sustainable Enhanced Catalytic Performance

**DOI:** 10.1038/srep08515

**Published:** 2015-02-17

**Authors:** Xiaobin Xie, Guanhui Gao, Zhengyin Pan, Tingjun Wang, Xiaoqing Meng, Lintao Cai

**Affiliations:** 1 Guangdong Key Laboratory of Nanomedicine, CAS Key Laboratory of Health Informatics, Institute of Biomedicine and Biotechnology, Shenzhen Institutes of Advanced Technology, Chinese Academy of Sciences, Shenzhen. 518055, P. R. China; 2 Nano Science and Technology Institute, University of Science & Technology of China, Suzhou. 215123, P. R. China

## Abstract

The catalytic activity of palladium (Pd) nanostructures highly relies on their size and morphology, especially enclosed with high-index facets, which provide more active sites so as to enhance their catalytic performance comparing with their low-index facet counterparts. Herein, Pd concave nanocubes enclosed with {730} facets by a one-pot scalable liquid method, with various high-index facets are synthesized via tuning reduction kinetics. Due to their high-index facets, the Pd concave nanocubes exhibit much higher electrocatalytic activity and stability for methanol oxidation than the Pd nanocubes enclosed by {100} facets and commercial Pd/C. Furthermore, we scale up synthesis of Pd concave nanocubes by expanding the volume of all species to fifty times with high-yield production.

Palladium (Pd) nanostructures with a variety of size and shape have been actively received great attention for years, due to their excellent performance as catalysts used in chemical industry, environmental technology and energy field^1^,^2^,^3^,^4^,^5^,^6^. Recent studies proposed that the catalytic activity was highly dependent on the size and morphology of Pd nanostructures, especially, the morphology played a significant role in determining the selectivity and active sites^7^,^8^,^9^,^10^. Very recently, Pd nanocrystals have been prepared with various morphologies including cube, octahedron, decahedron, icosahedrons and plate^11^,^12^,^13^,^14^,^15^. Most of them were enclosed by low-index facets such as {100}, {110} and {111}^12^,^13^,^14^,^15^,^16^,^17^. However, Pd nanostructures with high-index facets have generally exhibited much higher catalytic performance comparing with their low-index facet counterparts^10^,^18^,^19^,^20^,^21^. Moreover, synthesis of Pd nanocrystals enclosed by high-index facets has been challenged in engineering both size and morphology. Xia and his co-workers reported seed-mediated method for synthesis of Pd concave nanocubes (PdCNs) covered by high-index {730} facets using poly (vinylpyrrolidone) (PVP) and KBr as capping agents^22^. Zhang et al demonstrated a liquid approach, which reduced Na_2_PdCl_4_ by L-ascorbic acid (AA) and capped by cetyltrimethyl-ammonium bromide (CTAB) and cetyltrimethylammonium chloride (CTAC), towards preparation of Pd concave nanocubes enclosed by various high-index facets such as {730} and {310} facets^23^. It was reported that a Cu (II)-assisted seed-mediated protocol triggering Pd concave nanocubes^24^. Nonetheless, these synthesis processes of PdCNs were multi-stepped and impure products, which made it difficult to achieve large scalable yield^25^. Therefore, it is a great challenge to explore a robust and simple technique approaching a large yield with high-index enclosed facets.

Herein, we develop a scalable strategy to synthesize PdCNs with high-index facets via tuning reduction kinetics. It achieves the insights into high-volume production and controllable morphologies by manipulating the reaction temperature and the concentration of CTAB and AA. Generally, elevating the reaction temperature is beneficial to the formation of thermodynamic favored production—Pd nanocubes. While, the high-index facets PdCNs could be obtained by increasing concentration of AA but reducing the reaction temperature. Hence, the evolution of controllable Pd morphologies from nanocubes structures to various concave nanocubes is conducted by a series of optimization routine. Furthermore, we scale up synthesis of Pd concave nanocubes by approaching the volume of all species to fifty times as shown in scheme S1(Experimental section details in supporting information), which is in high-yield production of PdCNs. As expected, these PdCNs with high-index facets exhibit a highly enhanced catalytic property toward methanol oxidation comparing with the normal Pd nanocubes enclosed by low-index {100} facets, and commercial Pd/C. The success of exploring large yield PdCNs with high-index facets to improve catalytic performance would upgrade the pathway for designing more efficient catalysts.

## Results

**Figure 1:**
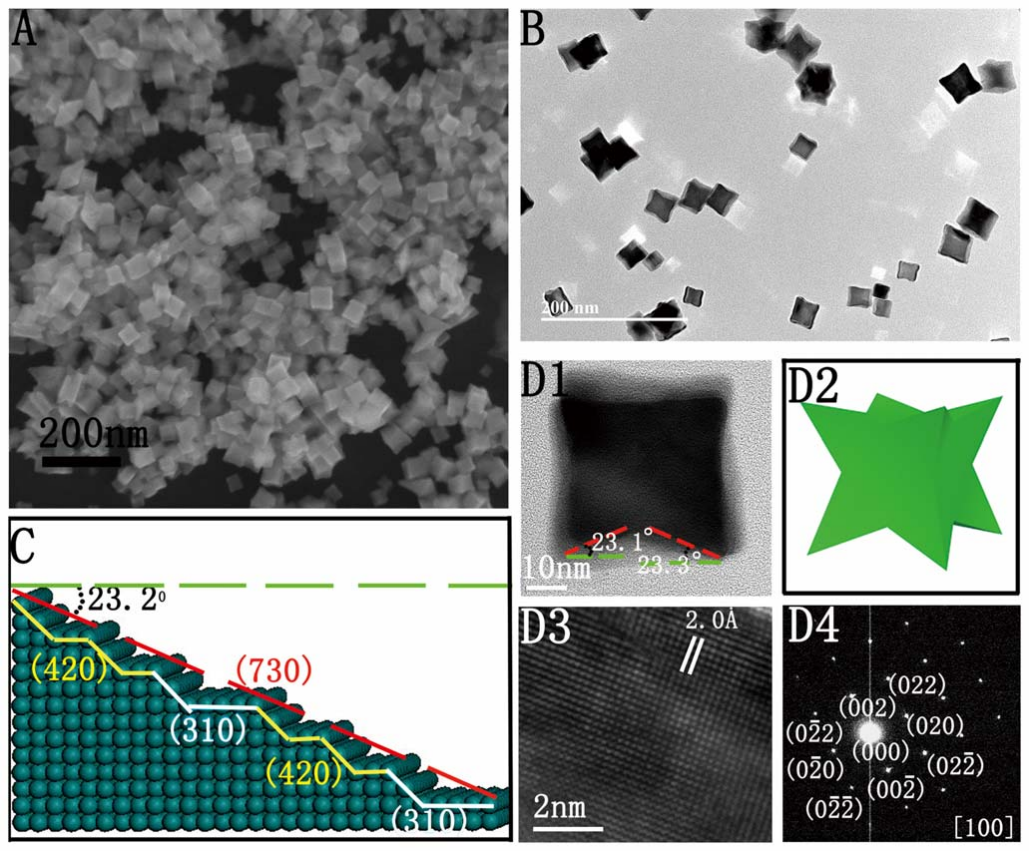
Morphology and lattice structure of Pd concave nanocubes (PdCNs). (A) SEM images of the PdCNs. (B) TEM image of PdCNs. (C) 3D model of high-index (730) facets. (D1, D3) high resolution bright-field TEM images of single PdCN. (D2) 3D model of PdCNs. (D4) selected area electron diffraction (SAED) pattern of PdCNs.

Figure 1A shows a typical field-emission scanning electron microscopy (FESEM) image of PdCNs synthesized using the protocol with high concentration of AA (Supporting Information Table S1–A3). The PdCNs as prepared are highly uniform with an average size of 30 nm. Transmission electron microscopy (TEM) is carried out for further characterization of the PdCNs. As shown by the TEM image (Figure 1B), each Pd concave nanocube exhibits a darker center compared to the edges, thus revealing the formation of concave structure basing on the particle surface. Figure 1 D1 demonstrates the TEM image of single PdCN along [001] zone axis, and the corresponding geometric model of individual PdCN in Figure 1 D2. The Miller indices of these edge-on faces could be identified as the {730} facets via investigating the angle of PdCN and calculating the values for several {hkl} facets^26^,^27^. Figure 1 C exhibits the three-dimensional atomic model, illustrating that the high-index {730} planes consisting of {310} and {420} sub-facets. Additionally, the single PdCN captured with HRTEM (Figure 1 D3) and selected-area electron diffraction (SAED) pattern (Figure 1 D4) clearly reveals the single crystalline structure.

**Figure 2:**
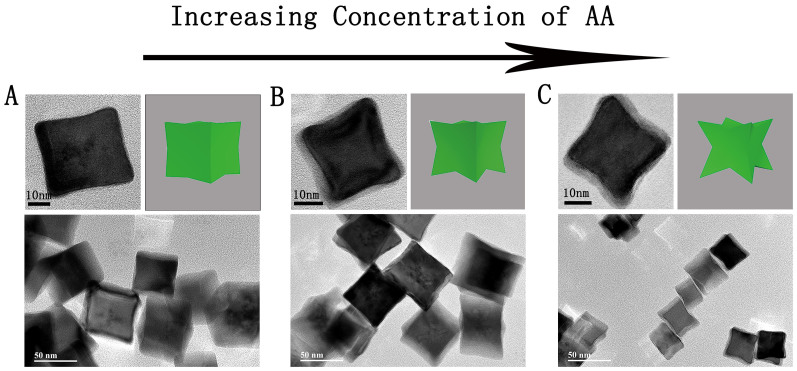
Morphology evolution of PdCNs via tuning the concentration of AA. The variation in concentration for AA listed below: (A) 3 mM; (B) 15 mM; (C) 60 mM.

A series of preparations are conducted via tuning temperature and concentration of AA and CTAB to further explore the morphology evolution and formation of PdCNs. Additional TEM images in Figure 2 and Figure S2 (Supporting Information) illustrate the slight change of PdCNs morphology with increasing the concentration of AA to a limited extent (Supporting Information Table S2). As shown in Figure 2, the PdCNs are obtained by using 3, 15 and 60 mM of AA respectively (Description in details seeing Supporting Information Table S1), correspondingly, the average size of PdCNs change seriously from 43 nm (Figure 2A and Figure S3 A), 38 nm (Figure 2B and Figure S3 B) to 30 nm (Figure 2 C and Figure S3 C). The morphology of PdCNs tends to be highly concave structure as the concentration of AA increasing. Therefore, the results indicate that both the shape and size of PdCNs are dramatically influenced by varying the concentration of AA. Similar to AA, CTAB plays a significant role for tailoring the morphology of PdCNs in this protocol. As shown in Figure S4, the PdCNs are prepared via tuning the concentration of CTAB from 2 to 50 mM and keeping same temperature (Supporting Information Table S3). As the consequence of CTAB variation, the Pd nanostructures emerge in various shapes simultaneously, including PdCNs, Pd nanorods (PdNRs), Pd tetrahedrons (PdTs) and other irregular nanoparticles (Figure S4). To sum up, the morphology of PdCNs could be tailored by varying the concentration of AA and CTAB to control the reaction kinetics.

For the synthesis of PdCNs with high-index facets controlled, reduction kinetics have an significant effect on manipulating nucleation and growth of nanocrystals^28^. In this study, both AA and CTAB are critical factors for the morphology evolution of Pd nanostructures. Being a one-pot protocol, the rapid depletion of reactants leading to nucleation and all subsequent growth occur at the pre-existing nuclei^29^. In this system, the size of PdCNs mostly depends on the numbers of nucleus. As the concentration of AA increasing, the reduction rate is accelerating dramatically, triggering the rapid growth of crystal nucleus, thus the size of PdCNs declined. For the morphology evolution, the high concentration of AA or low concentration of Br^−^ contributed from CTAB would induce the Pd nanostructures overgrowth at corners and edges^10^. Consequently, the PdCNs appear higher concave structure. The case of CTAB increasing is quite different from that of AA. The concentration of [PdBr_4_]^2−^ rising with the increase of CTAB inspires to slow down the reduction rates during the Pd nanostructures growth. It results in emergence of the low energy facets such as {100}, {110} and {111}, also being maintained by capping of Br^−^30,31. In general, high concentration of AA or low concentration of CTAB could be both essential to the formation of PdCNs.

**Figure 3:**
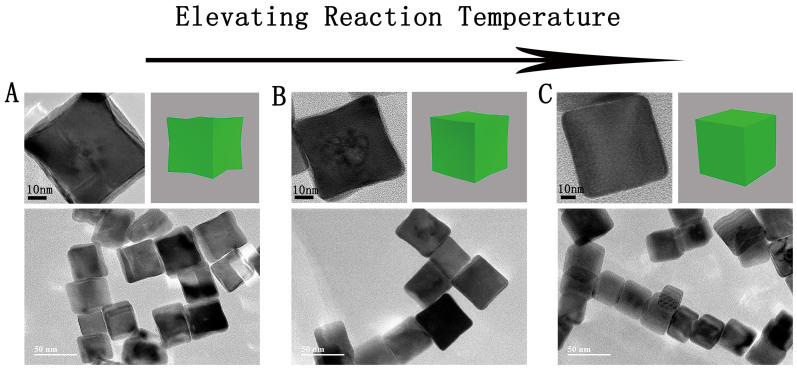
Morphology evolution of Pd nanostructures by elevating temperature, the temperature is controlled as follow: (A) 35 ~ 40°C; (B) 55 ~ 60°C; (C) 75 ~ 80°C.

In addition, the PdCNs are prepared by tuning different temperatures in order to investigate morphology variation (Table S4). As shown in Figure 3 and Figure S5, the products exhibit the shape evolution from PdCNs to Pd nanocubes as elevating the reduction temperature. At low temperature (35~40°C), the reaction tends to form PdCNs with average size of 43 nm (Figure 3A), while the products of PdCNs exhibit slight cube-like nanostructures with average size of 37 nm (Figure 3B) as elevating temperature range of 55~60°C. Finally, the products appear to be Pd nanocubes completely with 29 nm (Figure 3C) length at high temperature from 75~80°C. Above variation of morphologies is caused by the different surface energies of their structures. Furthermore, PdCNs encloseted by high-index facets possess much higher surface energy, so as to difficult maintain concave structure in the condition of high temperature. By Contrast, Pd nanocubes only expose low-index facets of {100}, which make them with lower surface energy and to be thermodynamic favor products^12^. Additionally, the reason of size variation is related to the reduction rate increase and the quantities of Pd nucleation as temperature rise^32^. At high temperature, the products come to being thermodynamic favor shape as Pd nanocubes and shorter average length of edge. It has been perplexing researchers for years in the view of scale-up synthesis of noble metal nanostructures^25^. For this purpose, we execute the preparation by expanding all species to fifty times (Table S5). The characterization and morphology of products are investigated using SEM and TEM (Figure S6). Most of Pd nanostructures still maintain concave shape with size similar to the small volume synthesis of PdCNs, and it is in high-yield production. These results illustrate that the scale-up strategy for synthesizing large volume PdCNs is feasible, which is significant for catalytic function applications at low cost and large production.

**Figure 4:**
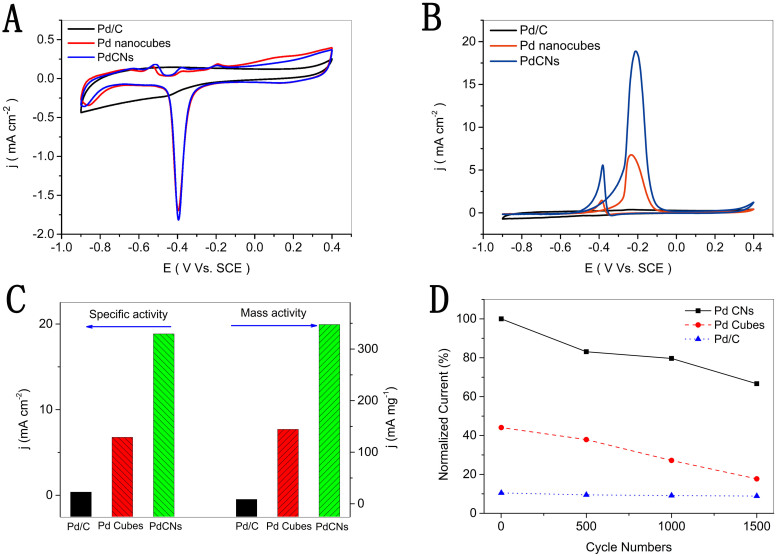
Cyclic Voltammograms of the PdCNs, Pd nanocubes and commercial Pd/C, which are recorded at room temperature with the scan rate of 50 mV/s in (A) 1M KOH solution; (B) 1M KOH contained 1M CH_3_OH; (C) The mass and specific activities (at −0.2 Vs.SCE); (D) Normalized current of 1500 cycles.

The PdCNs 30 nm in size with high-index {730} facets exposed on surface serve as catalysts for methanol electrooxidation. Figure 4 A demonstrates the cyclic votammetry (CV) profiles of the PdCNs, Pd nanocubes and commercial Pd/C in 1M KOH solution at room temperature, both PdCNs and Pd nanocubes exhibit higher intensity redox peaks than commercial Pd/C catalyst for comparison. The cathodic peak which is associated with the oxidation/reduction of Pd nanostructures, appears near 0.4 V versus saturated calomel electrode (SCE) for PdCNs and Pd nanocubes. Furthermore, the electrochemically active surface area (ECSA) of PdCNs, Pd nanocubes and commercial Pd/C is evaluated respectively by investigating the electric charges of oxygen desorption based on the CV curves recorded in the 0.5 M H_2_SO_4_ (Figure S7 A). There is a clear desorption peak for all samples near 0.4 V corresponding to the terrace on Pd surface. The calculated ECSA of PdCNs with 30 nm length is 18.472 m^2^/g, which is about 1.5 times and 1.9 times of that of Pd nanocubes (12.354 m^2^/g) and commercial Pd/C (9.651 m^2^/g), respectively (Figure S7 B). Notably, the ECSA of PdCNs with 43 nm length is 7.912 m^2^/g only, due to their smaller specific surface area comparably.

The higher oxidation/reduction activity of the PdCNs indicates their superior performance for the electrooxidation of methanol. Methanol oxidation measurements are further carried out in a solution containing 1M KOH and 1M methanol. As demonstrated in Figure 4B & C, the forward anodic peak current density for Pd catalyst increases in the order of Pd/C < Pd nanocubes < PdCNs, what is worth mentioning, the current density of that commercial Pd/C is about 0.374 mA/cm^2^, illustrating its weak catalytic property. And the catalytic current of PdCNs is greatly improved 2.8 times and 50 times compared to that of Pd naoncubes and Pd/C, respectively (Figure 4C). It manifests that the catalytic activity of PdCNs for methanol oxidation is significantly higher than Pd nanocubes and Pd/C. In addition, it is considered that the reverse-scan peaks are related to the tolerance of catalyst to the accumulation of intermediate carbonaceous species. Moreover, The PdCNs with more highly concave structure depression emerge enhanced catalytic activity (Figure S8), caused by abundant terraces and steps on surface. Furthermore, the accelerated CV measurements are performed to evaluate the catalytic stability. The peak current density of PdCNs still remain 66.62% after 1500 cycles as shown in Figure 4D, which is much higher than the Pd nanocubes and Pd/C comparably. Additionally, TEM characterization confirms that most of PdCNs maintain their concave structures after 1500 cycles measurement (Figure S9 A–D), evidencing their long-term durability.

In summary, we develop a one-pot strategy with large-scale yield to synthesize of PdCNs enclosed by high-index 24 {730} facets. The conditions of low temperature and high concentration of AA are both favored to induce to well-defined concave structure. The PdCNs have plenty steps, corners and edge-sites triggering more active sites, which sustainably enhance higher catalytic performance and durability for methanol oxidation than Pd nanocubes and commercial Pd/C. More importantly, this facile approach provides a bright prospect for achieving large-scale preparation of noble metal nanostructures with their morphologies controlling simultaneously.

## Methods

### Preparation of Pd concave nanocubes

Typically, mixture of 0.1 mL of 0.1 M CTAB, 4.4 mL of deionized water and 0.5 mL of 10 mM K_2_PdCl_4_ in a 10 mL bottle, then added 0.15 mL of 0.1 M AA into above mixture with magnetic stirring. The bottle was shifted in 35~40°C water bath under magnetic stirring. The black product was collected by centrifugation at 12000 rpm for 10 min, washed by deionized water for several times to removal excess CTAB. (Details and conditions change show as Table S1 to Table S4)

### Large-scale preparation of Pd concave nanocubes

The protocol was similar to the typical synthesis, except that expand the amount of all species to fifty times (Details show as Table S5.).

### Electrochemical Measurement

The electrochemical activities of Pd nanocatalysts were performed at room temperature using a three-electrode system consisting a glassy carbon electrode (GCE, 5 mm diameter, geometric area of 0.196 cm^2^), a Pt plate (1 × 1 cm^2^) counter electrode, and a saturated calomel electrode (SCE) at an electrochemical station (CHI660E). Representatively, 0.6 mg of Pd nano-catalyst and 150 μL of Nafion solution (0.1 wt%) were dispersed in 300 μL of water-ethanol solution with volume ratio of 3:1 followed by ultra-sonication for 0.5 h to form a homogeneous ink. Then 15 μL of the dispersion (containing 20 μg of catalyst) was loaded onto the GCE (loading catalyst 0.102 mg cm^−2^). Prior to test, the solution of 1.0 M KOH and 1.0 M CH_3_OH was purged with pure Ar gas for 1 h. Methanol oxidation measurements were conducted in a solution containing 1.0 M KOH and 1.0 M CH_3_OH using GCE at a sweep rate of 50 mV/s. The stability test was performed at a sweep rate of 0.5 V/s in a 1.0 M KOH and 1.0 M CH_3_OH solution for 1500 cycles.

### Characterizations

Transmission electron microscopy (TEM), high resolution bright-field TEM, and selected area electron diffraction (SAED) measurements were carried out with the field emission FEI-F20, operated at 200 kV.

## Supplementary Material

Supplementary Information Supporting informaion

## References

[b1] Huang X. *et al.* Palladium-based nanostructures with highly porous features and perpendicular pore channels as enhanced organic catalysts. Angew. Chem. Int. Edit. 52, 2520–2524 (2013). 10.1002/anie.201208901 23362177

[b2] Ji X. *et al.* Nanocrystalline intermetallics on mesoporous carbon for direct formic acid fuel cell anodes. Nat. Chem. 2, 286–293 (2010). 21124509 10.1038/nchem.553

[b3] Kesavan L. *et al.* Solvent-free oxidation of primary carbon-hydrogen bonds in toluene using Au-Pd alloy nanoparticles. Science 331, 195–199 (2011). 21233383 10.1126/science.1198458

[b4] Tedsree K. *et al.* Hydrogen production from formic acid decomposition at room temperature using a Ag-Pd core-shell nanocatalyst. Nat. Nanotechnol. 6, 302–307 (2011). 21478867 10.1038/nnano.2011.42

[b5] Xu C. W., Wang H., Shen P. K. & Jiang S. P. Highly ordered Pd nanowire arrays as effective electrocatalysts for ethanol oxidation in direct alcohol fuel cells. Adv. Mater. 19, 4256–4259 (2007).

[b6] Antolini E. Palladium in fuel cell catalysis. Energy Environ. Sci. 2, 915 (2009).

[b7] Zhou W. P. *et al.* Size effects in electronic and catalytic properties of unsupported palladium nanoparticles. J. Phys. Chem. B 10, 13393–13398 (2006). 10.1021/jp061690h 16821860

[b8] Zhou Z. Y., Tian N., Li J. T., Broadwell I. & Sun S. G. Nanomaterials of high surface energy with exceptional properties in catalysis and energy storage. Chem. Soc. Rev. 40, 4167–4185 (2011). 21552612 10.1039/c0cs00176g

[b9] Yang C. W. *et al.* Fabrication of Au-Pd core-shell heterostructures with systematic shape evolution using octahedral nanocrystal cores and their catalytic activity. J. Am. Chem. Soc. 133, 19993–20000 (2011). 22091631 10.1021/ja209121x

[b10] Zhang H., Jin M. & Xia Y. Noble-metal nanocrystals with concave surfaces: synthesis and applications. Angew. Chem. Int. Edit. 51, 7656–7673 (2012). 10.1002/anie.201201557 22639064

[b11] Xiong Y. & Xia Y. Shape-controlled synthesis of metal nanostructures: the case of palladium. Adv. Mater. 19, 3385–3391 (2007).

[b12] Xia Y., Xiong Y., Lim B. & Skrabalak S. E. Shape-controlled synthesis of metal nanocrystals: simple chemistry meets complex physics? Angew. Chem. Int. Edit. 48, 60–103 (2009). 10.1002/anie.200802248 PMC2791829 19053095

[b13] Chen Y. H., Hung H. H. & Huang M. H. Seed-mediated synthesis of palladium nanorods and branched nanocrystals and their use as recyclable suzuki coupling reaction catalysts. J. Am. Chem. Soc. 131, 9114–9121 (2009). 19507854 10.1021/ja903305d

[b14] Mazumder V. & Sun S. Oleylamine-mediated synthesis of Pd nanoparticles for catalytic formic acid oxidation. J. Am. Chem. Soc. 131, 4588–4589 (2009). 19281236 10.1021/ja9004915

[b15] Xiong Y. *et al.* Synthesis and mechanistic study of palladium nanobars and nanorods. J. Am. Chem. Soc. 129, 3665–3675 (2007). 17335211 10.1021/ja0688023

[b16] Xia X. *et al.* Facile synthesis of palladium right bipyramids and their use as seeds for overgrowth and as catalysts for formic acid oxidation. J. Am. Chem. Soc. 135, 15706–15709 (2013). 24106797 10.1021/ja408018j

[b17] Zhang J. *et al.* Shape-controlled synthesis of palladium single-crystalline nanoparticles: the effect of HCl oxidative etching and facet-dependent catalytic properties. Chem. Mater. 26, 1213–1218 (2014).

[b18] Feldheim D. L. Chemistry. the new face of catalysis. Science 316, 699–700 (2007). 17478708 10.1126/science.1143093

[b19] Tian N., Zhou Z. Y., Yu N. F., Wang L. Y. & Sun S. G. Direct electrodeposition of tetrahexahedral Pd nanocrystals with high-index facets and high catalytic activity for ethanol electrooxidation. J. Am. Chem. Soc. 132, 7580–7581 (2010). 20469858 10.1021/ja102177r

[b20] Xia B. Y., Wu H. B., Wang X. & Lou X. W. Highly concave platinum nanoframes with high-index facets and enhanced electrocatalytic properties. Angew. Chem. Int. Edit. 52, 12337–12340 (2013). 10.1002/anie.201307518 24115319

[b21] Zhang L., Niu W. & Xu G. Synthesis and applications of noble metal nanocrystals with high-energy facets. Nano Today 7, 586–605 (2012).

[b22] Jin M., Zhang H., Xie Z. & Xia Y. Palladium concave nanocubes with high-index facets and their enhanced catalytic properties. Angew. Chem. Int. Edit. 50, 7850–7854 (2011). 10.1002/anie.201103002 21732512

[b23] Zhang J. *et al.* Synthesis of concave palladium nanocubes with high-index surfaces and high electrocatalytic activities. Chem. Eur. J. 17, 9915–9919 (2011). 21805511 10.1002/chem.201100868

[b24] Niu W., Zhang W., Firdoz S. & Lu X. Controlled synthesis of palladium concave nanocubes with sub-10-nanometer edges and corners for tunable plasmonic property. Chem. Mater. 26, 2180–2186 (2014).

[b25] Zhang H., Jin M., Xiong Y., Lim B. & Xia Y. Shape-controlled synthesis of Pd nanocrystals and their catalytic applications. Acc. Chem. Res. 46, 1783–1794 (2013). 23163781 10.1021/ar300209w

[b26] Tian N., Zhou Z. Y., Sun S. G., Ding Y. & Wang Z. L. Synthesis of tetrahexahedral platinum nanocrystals with high-index facets and high electro-oxidation activity. Science 316, 732–735 (2007). 17478717 10.1126/science.1140484

[b27] Tian N., Zhou Z. Y. & Sun S. G. Platinum metal catalysts of high-index surfaces from single-crystal planes to electrochemically shape-controlled nanoparticles. J. Phys. Chem. C. 112, 19801–19817 (2008).

[b28] Lim B. *et al.* Shape-controlled synthesis of Pd nanocrystals in aqueous solutions. Adv. Funct.Mater. 19, 189–200 (2009).

[b29] Tao A. R., Habas S. & Yang P. Shape control of colloidal metal nanocrystals. Small 4, 310–325 (2008).

[b30] Liu M., Zheng Y., Zhang L., Guo L. & Xia Y. Transformation of Pd nanocubes into octahedra with controlled sizes by maneuvering the rates of etching and regrowth. J. Am. Chem. Soc. 135, 11752–11755 (2013). 23902400 10.1021/ja406344j

[b31] Peng H. C., Xie S., Park J., Xia X. & Xia Y. Quantitative analysis of the coverage density of Br-ions on Pd{100} facets and its role in controlling the shape of Pd nanocrystals. J. Am. Chem. Soc. 135, 3780–3783 (2013). 23438500 10.1021/ja400301k

[b32] Xiong Y. *et al.* Kinetically controlled synthesis of triangular and hexagonal nanoplates of palladium and their SPRSERS properties. J. Am. Chem. Soc. 127, 17118–17127 (2005). 16316260 10.1021/ja056498s

