# Supplementary material for: Large-Scale Synthesis of Palladium Concave Nanocubes with High-Index Facets for Sustainable Enhanced Catalytic Performance
Source: Sci Rep. 2015 Feb 17;5:8515. doi: 10.1038/srep08515 (PMC4330526; doi:10.1038/srep08515)
Supplement: Supplementary Information — Supporting informaion [file srep08515-s1.docx]

Supporting Information

**Large-scale Synthesis of Palladium Concave Nanocubes with High-index Facets for Sustainable Enhanced Catalytic Performance**

**Xiaobin Xie^a,b‡^, Guanhui Gao^a‡^, Zhengyin Pan^a^, Tingjun Wang^b^, Xiaoqing Meng^a,b^, and Lintao Cai^a,^***

**1 Materials and Methods**

**1.1 Materials:** Potassium tetrachchloropalladium (II) (K_2_PdCl_4_, 99.95%, J&K), L-ascorbic acid (AA, 99.98%, J&K), cetyltrimethyl-ammonium bromide(CTAB, ≥99%, Sigma), Potassium Hydroxide(KOH, ≥88%, Shanghai Lingfeng Reagent Company), methanol (≥99.5%, Shanghai Lingfeng Reagent Company), Ethanol (99.7%, Shanghai Lingfeng Reagent Company), the deionized water with a resistivity of 18.2 MΩ∙cm was used in the experiment.

**1.2 Methods:** Transmission electron microscopy (TEM), high resolution bright-field TEM, and selected area electron diffraction (SAED) measurements were carried out with the field emission FEI-F20, operated at 200 kV.

**2. Experimental section**

**2.1 Preparation of Pd concave nanocubes:** Typically, mixture of 0.1 mL of 0.1 M CTAB, 4.4mL of deionized water and 0.5 mL of 10 mM K_2_PdCl_4_ in a 10 mL bottle, then added 0.15mL of 0.1 M AA into above mixture with magnetic stirring. The bottle was shifted in 35～40 ℃ water bath under magnetic stirring. The black product was collected by centrifugation at 12000 rpm for 10 min, washed by deionized water for several times to removal excess CTAB.( Details and conditions change show as **Table S1** to **Table S4**)

**2.2 Large-scale preparation of Pd concave nanocubes:** The protocol was similar to the typical synthesis, except that expand the amount of all species to fifty times (Details show as **Table S5**.).

**2.3 Electrochemical Measurement:** The electrochemical activities of Pd nanocatalysts were performed at room temperature using a three-electrode system consisting a glassy carbon electrode (GCE, 5 mm diameter, geometric area of 0.196 cm^2^), a Pt plate (1×1 cm^2^) counter electrode, and a saturated calomel electrode (SCE) at an electrochemical station (CHI660E). Representatively, 0.6 mg of Pd nanocatalyst and 150 µL of Nafion solution (0.1wt%) were dispersed in 300 µL of water-ethanol solution with volume ratio of 3:1 followed by ultra-sonication for 0.5 h to form a homogeneous ink. Then 15 µL of the dispersion (containing 20 µg of catalyst) was loaded onto the GCE (loading catalyst 0.102 mg cm^-2^). Prior to test, the solution of 1.0 M KOH and 1.0 M CH_3_OH was purged with pure Ar gas for 1h.Methanol oxidation measurements were conducted in a solution containing 1.0 M KOH and 1.0 M CH_3_OH using GCE at a sweep rate of 50 mV/s. The stability test was performed at a sweep rate of 0.5 V/s in a 1.0 M KOH and 1.0 M CH_3_OH solution for 1500 cycles.

**Table S1.** Protocol of prepare Pd concave nanocubes via tuning the concentration of AA from 3 mM to 60 mM

|  | A1 | A2 | A3 |
| --- | --- | --- | --- |
| H_2_O / ml | 4.25 | 3.65 | 1.40 |
| 0.1M CTAB / ml | 0.10 | 0.10 | 0.10 |
| 0.01M K_2_PdCl_4_ / ml | 0.50 | 0.50 | 0.50 |
| 0.1M AA / ml | 0.15 | 0.75 | 3.00 |
| Temperature / ℃ | 35~40 | 35~40 | 35~40 |

**Table S2.** Protocol of prepare Pd concave nanocubes via tuning the concentration of AA from 2 mM to 8 mM

|  | A1 | A2 | A3 | A4 |
| --- | --- | --- | --- | --- |
| H_2_O / ml | 4.3 | 4.2 | 4.1 | 4.0 |
| 0.1M CTAB / ml | 0.1 | 0.1 | 0.1 | 0.1 |
| 0.01M K_2_PdCl_4_ / ml | 0.5 | 0.5 | 0.5 | 0.5 |
| 0.1M AA / ml | 0.1 | 0.2 | 0.3 | 0.4 |
| Temperature / ℃ | 35~40 | 35~40 | 35~40 | 35~40 |

**Table S3.** The details of prepare Pd nanostructures via tuning the concentration of CTAB from 2 mM to 50 mM

|  | C1 | C2 | C3 | C4 |
| --- | --- | --- | --- | --- |
| H_2_O / ml | 4.25 | 3.85 | 3.35 | 1.85 |
| 0.1M CTAB / ml | 0.10 | 0.50 | 1.00 | 2.50 |
| 0.01M K_2_PdCl_4_ / ml | 0.50 | 0.50 | 0.50 | 0.50 |
| 0.1M AA / ml | 0.15 | 0.15 | 0.12 | 0.15 |
| Temperature / ℃ | 35~40 | 35~40 | 35~40 | 35~40 |

**Table S4.** The details of prepare Pd nanostructures at different temperature

|  | T1 | T2 | T3 |
| --- | --- | --- | --- |
| H_2_O / ml | 4.25 | 4.25 | 4.25 |
| 0.1M CTAB / ml | 0.10 | 0.10 | 0.10 |
| 0.01M K_2_PdCl_4_ / ml | 0.50 | 0.50 | 0.50 |
| 0.1M AA / ml | 0.15 | 0.15 | 0.15 |
| Temperature / ℃ | 35~40 | 55~60 | 75~80 |

**Table S5.** Protocol of prepare Pd concave nanocubes with large volume

|  | Typical Volume | Large Volume |
| --- | --- | --- |
| H_2_O / ml | 3.4 | 170.0 |
| 0.1M CTAB / ml | 0.1 | 5.0 |
| 0.01M K_2_PdCl_4_ / ml | 0.5 | 25.0 |
| 0.1M AA / ml | 1.0 | 50.0 |
| Total Volume / ml | 5.0 | 250.0 |
| Temperature / ℃ | 35~40 | 35~40 |


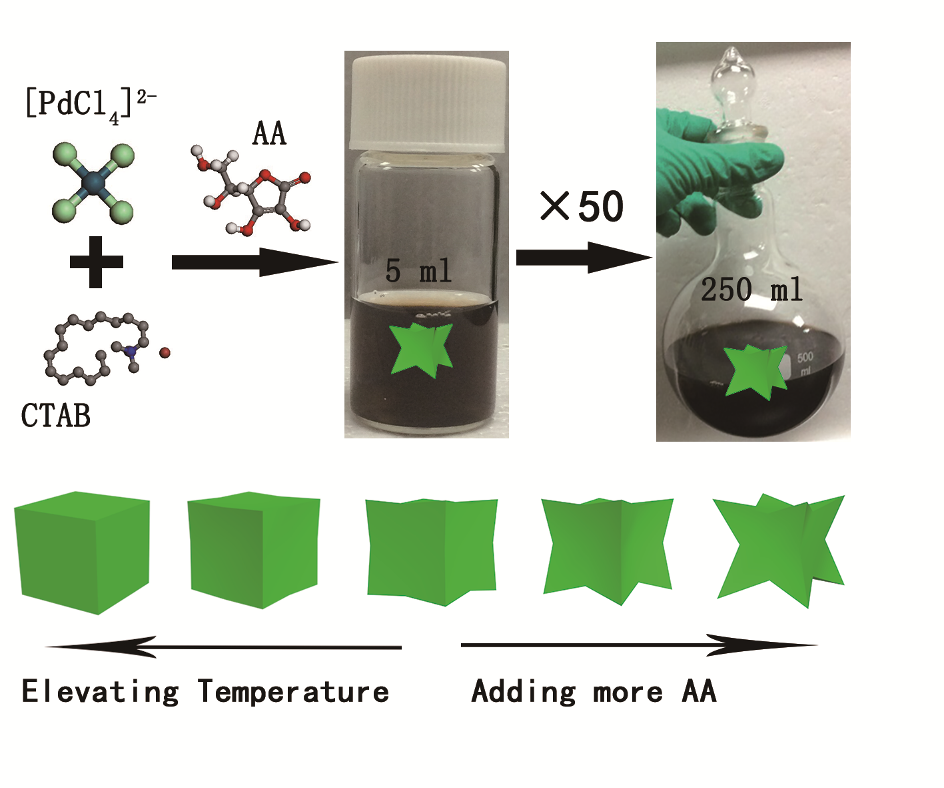


**Scheme S1︳**Schematic depiction of large-scale synthesis of palladium concave nanocubes with different morphologies triggered by AA and reaction temperature


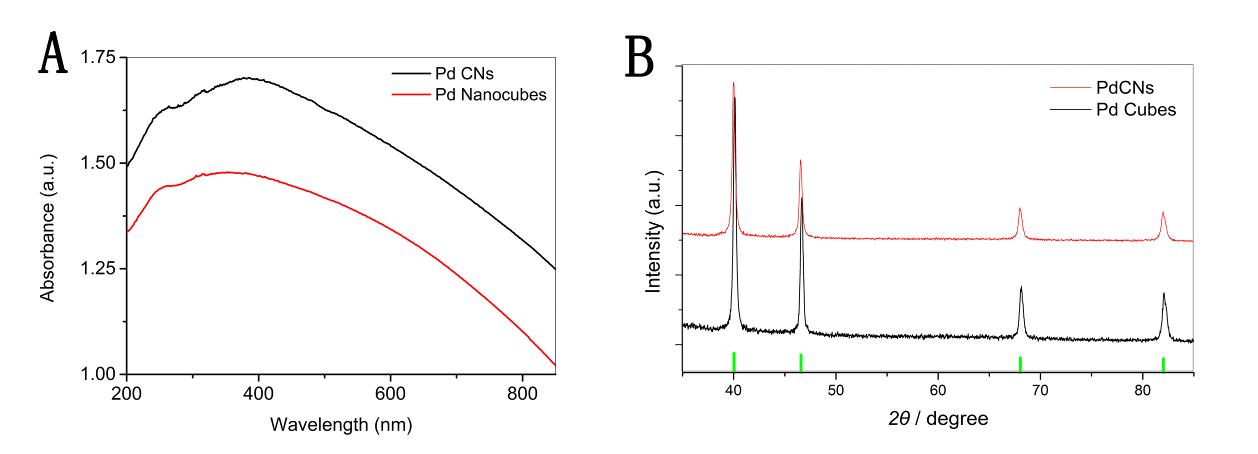


**Figure S1.** Uv-vis and XRD of PdCNs and Pd nanocubes. (A) Uv-Vis of PdCNs and Pd nanocubes ; (B) XRD pattern of PdCNs and Pd nanocubes. The green bar shows the calculation values for Pd (JCPDS No.46-10473).


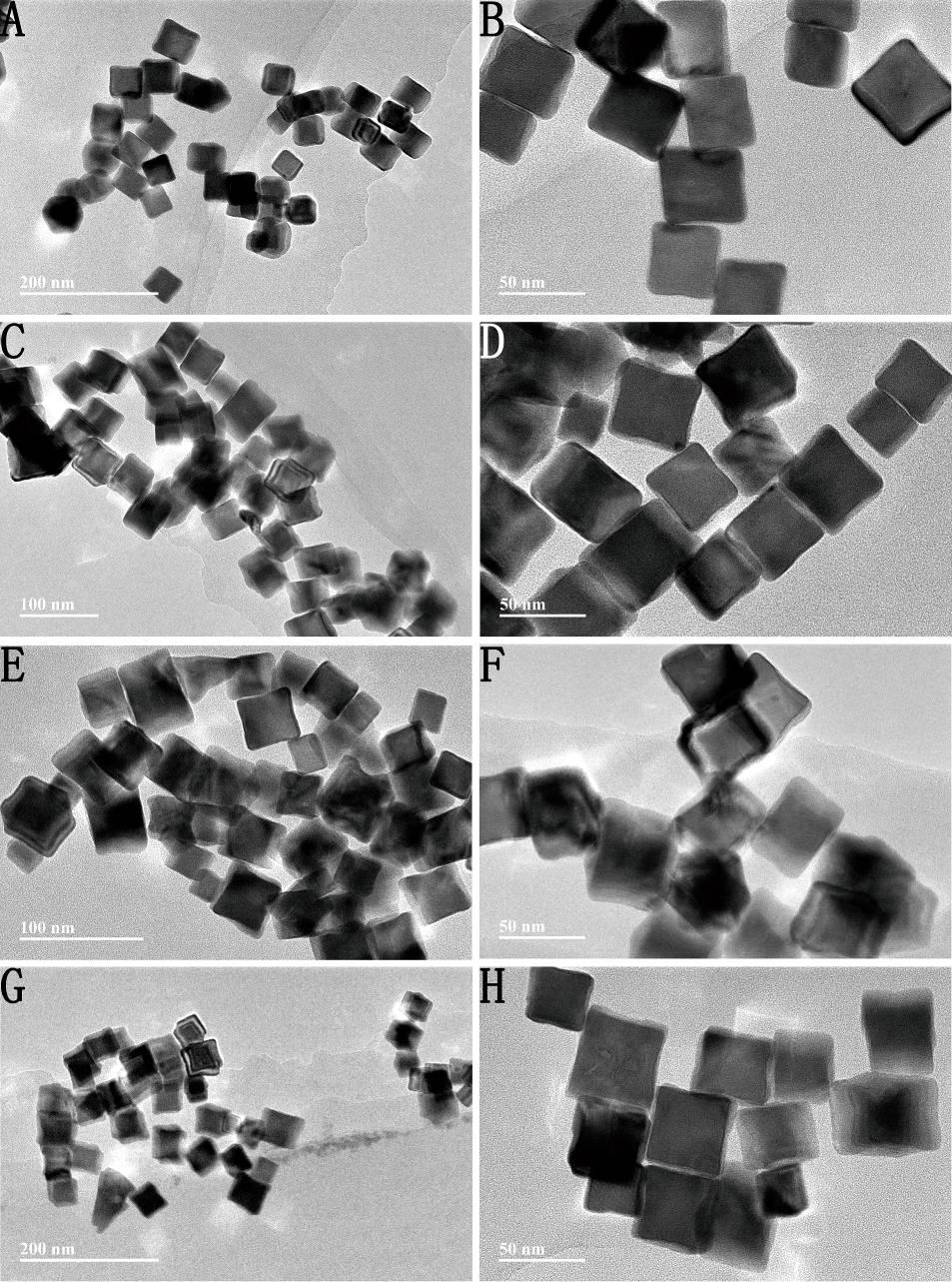


**Figure S2.** Morphology evolution of PdCNs via change the concentration of AA. The variation in concentration for AA listed below: (A & B) 2mM; (C & D) 4mM; (E & F) 6mM; (G & H) 8mM.


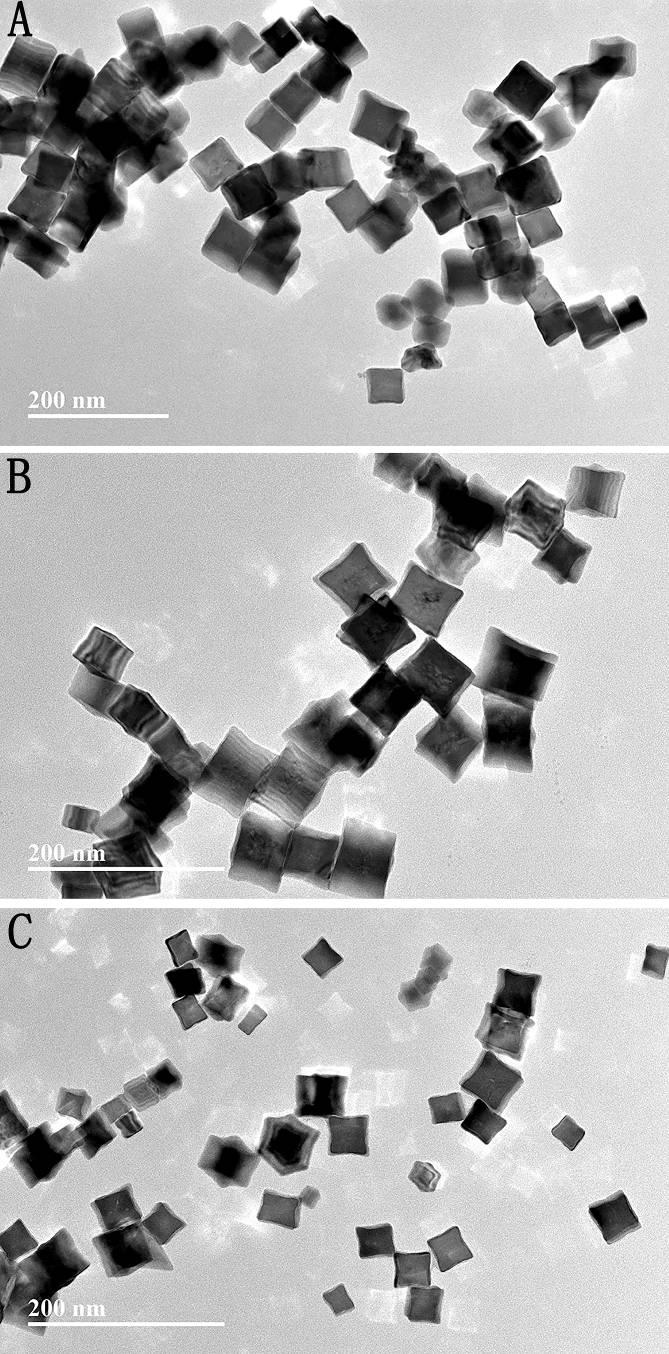


**Figure S3.** Morphology evolution of PdCNs via change the concentration of AA. The variation in concentration for AA listed below: (A) 3mM; (B) 15mM; (C) 60mM.


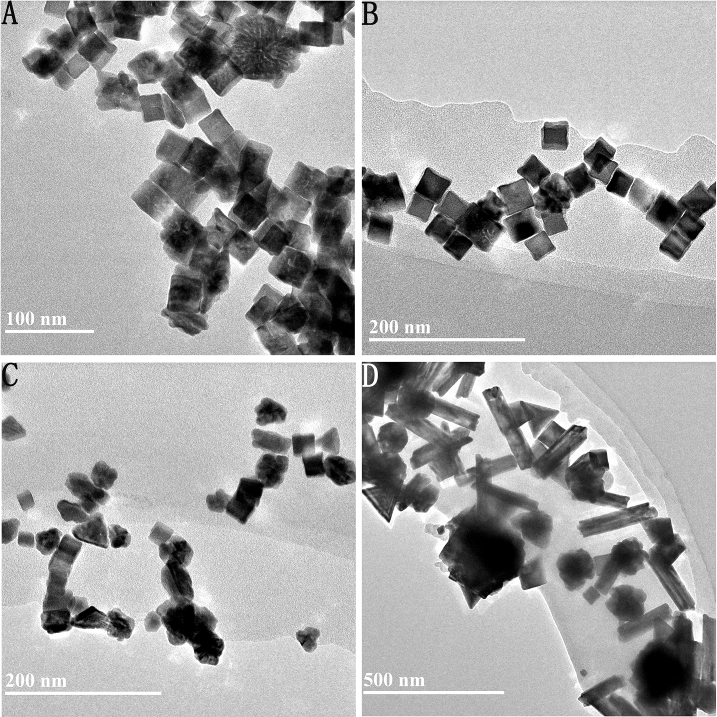


**Figure S4** Morphology evolution of Pd nanostructures via change the concentration of CTAB.The variation in concentration for CTAB listed below: (A) 2mM; (B) 10mM; (C) 20mM; (D) 50mM.

**
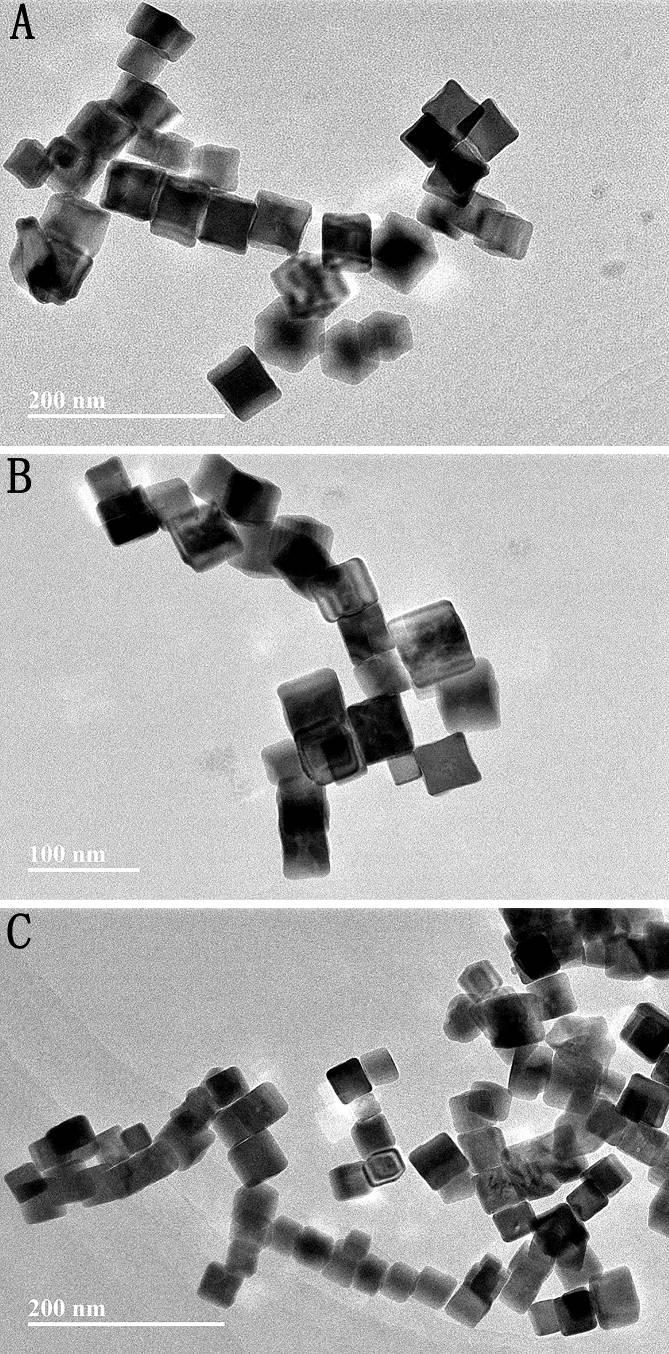
**

**Figure S5** Morphology evolution of Pd nanostructures by elevating temperature, the temperature is controlled as follow: (A) 35~40 ℃; (B) 55~60 ℃; (C) 75~80 ℃.


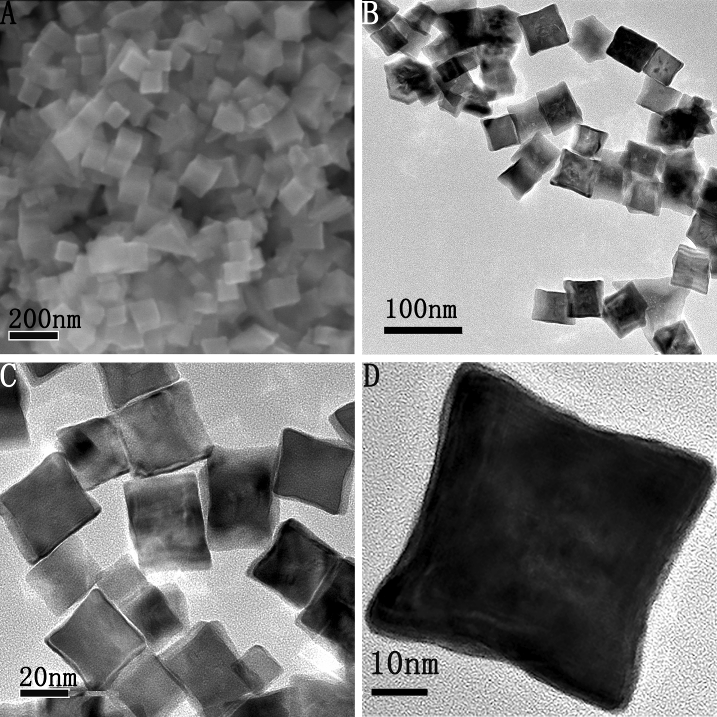


**Figure S6** Morphology of Pd concave nanocubes (PdCNs) which are prepared in high-yield production by expanding all species to fifty times. (A) SEM images of the PdCNs. (B, C) TEM image of PdCNs. (D) HRTEM image of PdCNs.


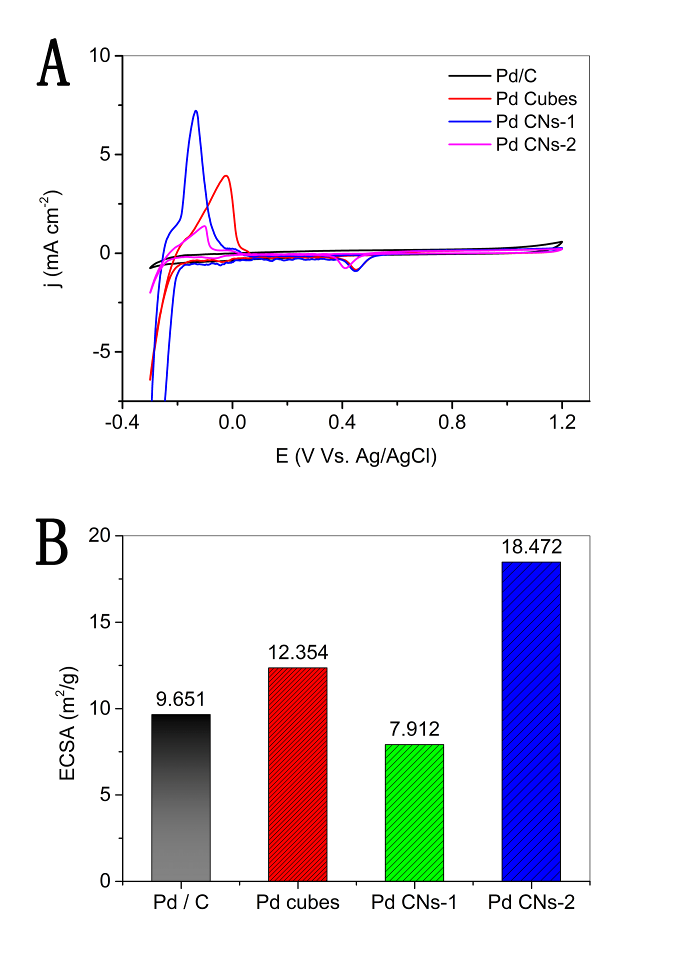


**Figure S7.** Cyclic Voltammograms of Pd/C, Pd nanocubes and PdCNs (PdCNs-1 is prepared as Table S1-A1, PdCNs-2 is prepared as Table S1-A3), which are recorded at room temperature with the scan rate of 50mV/s in (A) 0.5M H_2_SO_4_ solution and (B) Electrochemically active surface area (ECSA).


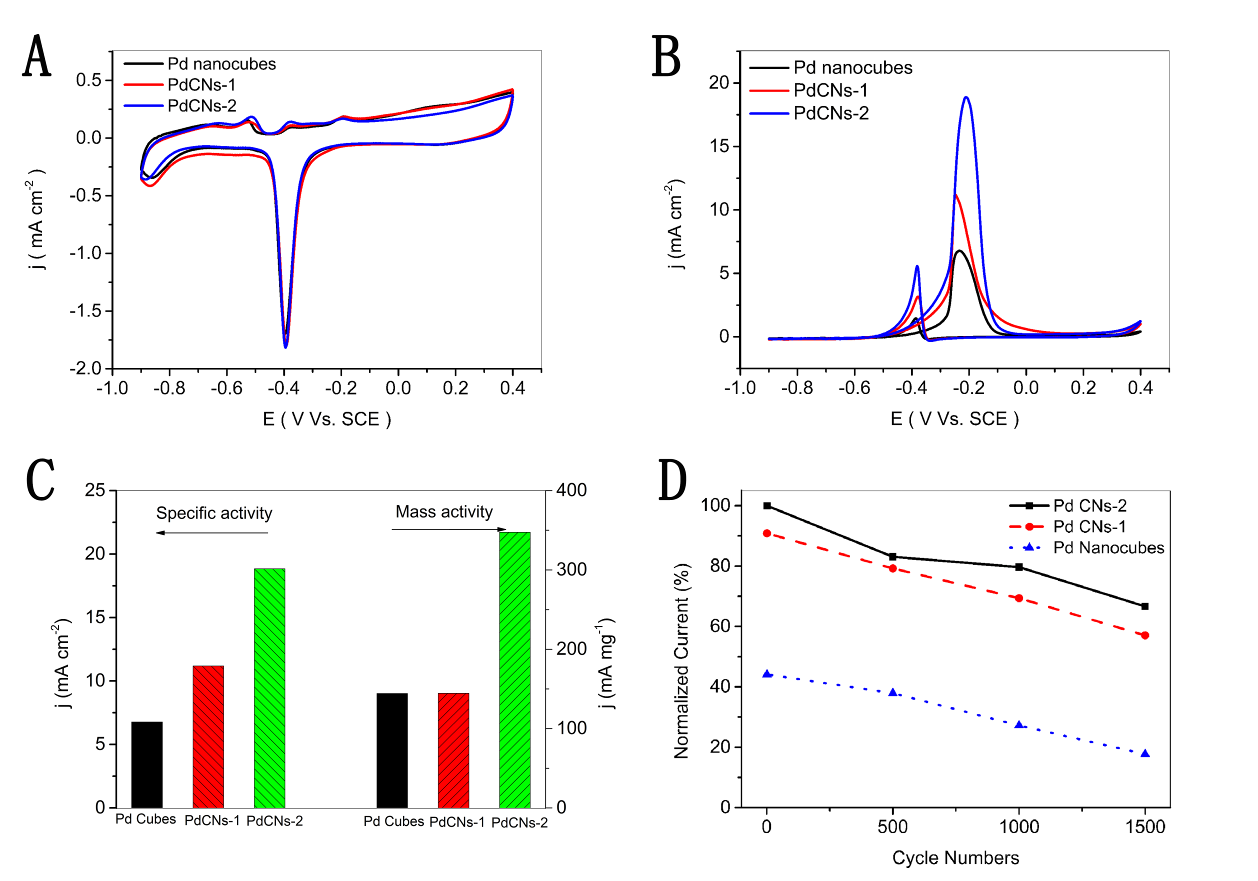


**Figure S8.** Cyclic Voltammograms of the Pd nanocubes and PdCNs (PdCNs-1 is prepared as Table S1-A1, PdCNs-2 is prepared as Table S1-A3), which are recorded at room temperature with the scan rate of 50 mV/s in (A) 1M KOH solution and (B) 1M KOH contained 1M CH_3_OH; (C) The mass and specific activities (at -0.2 Vs. SCE); (D) Normalized current of 1500 cycles.


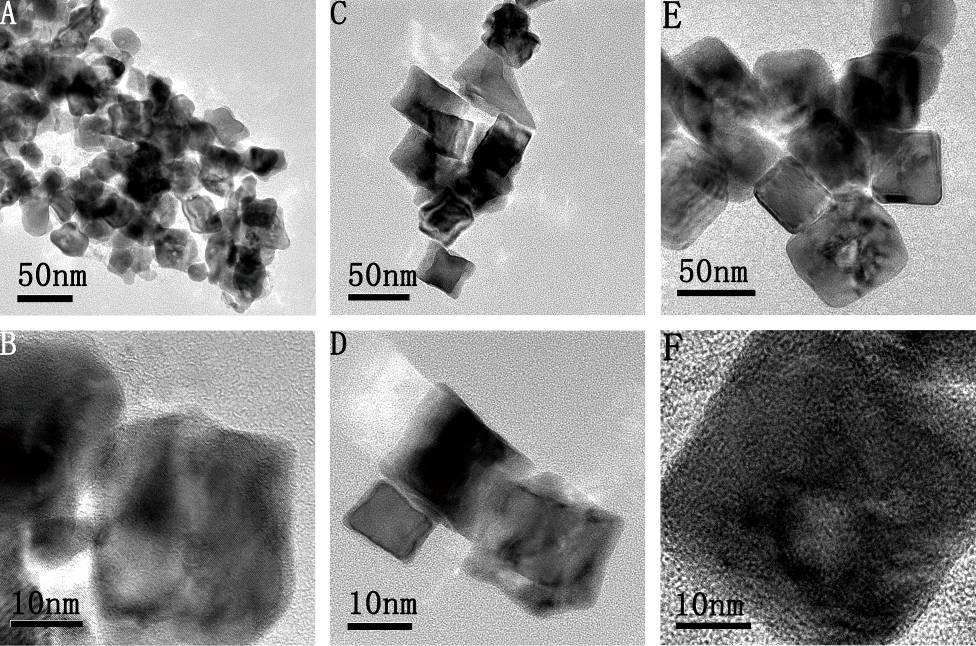


**Figure S9.** TEM images for morphology evolution of PdCNs (A-D) and Pd nanocubes (E, F) after 1500 cycles catalytic stability measurement.
